# Molecular cloning and functional expression of geranylgeranyl pyrophosphate synthase from *Coleus forskohlii *Briq

**DOI:** 10.1186/1471-2229-4-18

**Published:** 2004-11-18

**Authors:** Surang Engprasert, Futoshi Taura, Makoto Kawamukai, Yukihiro Shoyama

**Affiliations:** 1 Graduate School of Pharmaceutical Science, Kyushu University, 3-1-1 Maidashi, Higashi-ku, Fukuoka 812-8582, Japan; 2 Department of Applied Bioscience and Biotechnology, Faculty of Life and Environment Science, Shimane University, Matsue 690-8504, Japan

## Abstract

**Background:**

Isopentenyl diphosphate (IPP), a common biosynthetic precursor to the labdane diterpene forskolin, has been biosynthesised via a non-mevalonate pathway. Geranylgeranyl diphosphate (GGPP) synthase is an important branch point enzyme in terpenoid biosynthesis. Therefore, GGPP synthase is thought to be a key enzyme in biosynthesis of forskolin. Herein we report the first confirmation of the *GGPP synthase *gene in *Coleus forskohlii *Briq.

**Results:**

The open reading frame for full-length *GGPP synthase *encodes a protein of 359 amino acids, in which 1,077 nucleotides long with calculated molecular mass of 39.3 kDa. Alignments of *C. forskohlii GGPP synthase *amino acid sequences revealed high homologies with other plant *GGPP synthases*. Several highly conserved regions, including two aspartate-rich motifs were identified. Transient expression of the N-terminal region of *C. forskohlii *GGPP synthase-GFP fusion protein in tobacco cells demonstrated subcellular localization in the chloroplast. Carotenoid production was observed in *Escherichia coli *harboring pACCAR25Δ*crtE *from *Erwinia uredovora *and plasmid carrying *C. forskohlii GGPP synthase*. These results suggested that cDNA encoded functional GGPP synthase. Furthermore, *C. forskohlii GGPP synthase *expression was strong in leaves, decreased in stems and very little expression was observed in roots.

**Conclusion:**

This investigation proposed that forskolin was synthesised via a non-mevalonate pathway. GGPP synthase is thought to be involved in the biosynthesis of forskolin, which is primarily synthesised in the leaves and subsequently accumulates in the stems and roots.

## Background

Forskolin, a labdane diterpene, is a major active compound isolated from tuberous roots of *Coleus forskohlii *Briq. (Lamiaceae) [1]. *C. forskohlii *has been used as an important folk medicine in India. Futher, forskolin has been found to be a potent activator of adenylate cyclase [2], leading to an increase in levels of c-AMP, which affects heart action, blood and intraocular pressure. Recently, forskolin has become commercially available as a drug for treating heart disease in Japan. Forskolin is not available by chemical synthesis due to its complicated structure. However, two groups have reported successful total synthesis of forskolin [3,4].

Isoprenoids are essential for the normal growth and development processes in all living organisms. Isopentenyl diphosphate (IPP; C_5_) is a common metabolic precursor of all isoprenoids. Recently, several groups have demonstrated that two distinct pathways synthesise IPP in plants. The mevalonate (MVA) pathway occurs in the cytoplasm, and an alternative mevalonate-independent (2C-methyl-D-erythritol 4-phosphate; MEP) pathway occurs in plastids [5-7].

Geranylgeranyl diphosphate (GGPP) synthase catalyses the consecutive condensation of an allylic diphosphate with three molecules of IPP to produce GGPP, an essential linear precursor for biosynthesis of diterpenes, carotenoid, retinoids and side chain of chlorophyll [8]. GGPP synthase is an important branch point prenyltransferase enzyme in terpenoid biosynthesis.

*GGPP synthase *genes have been cloned in a number of organisms including; *Arabidopsis thaliana *[9,10], *Taxus canadensis *[11], *Helianthus annuus *[12], *Scoparia dulcis *and *Croton sublyratus *[13], *Sulfolobus acidocaldarius *[14], *Neurospora crassa *[15], and mouse and human [16]. Amino acid sequence comparison has shown that GGPP synthases contain several domains of conserved amino acid residues including the first aspartate-rich motifs (FARM) and the second aspartate-rich motif (SARM) [17]. Futhermore, recent studies suggested that two amino acids at the four and five positions before FARM in the sequence, as well as an insertion in FARM of plant GGPP synthases play important roles in product length determination [13,18].

Carotenoids arise from the coupling of two molecules of GGPP. The carotenoid biosynthetic gene cluster (*crt *genes) of *Erwinia uredovora *was elucidated [19], and is currently used to investigate the function of carotenoid related genes in a heterologous system. This *crt *gene cluster is composed of six genes; *crtB *(phytoene synthase), *crtE *(GGPP synthase), *crtI *(phytoene desaturase), *crtX *(zeaxanthin β-glucosidase), *crtY *(lycopene cyclase) and *crtZ *(β-carotene hydroxylase). Consequently, the production of carotenoids using *E. coli *harbouring the *crt *gene cluster can be used for the determination of GGPP synthase activity.

GGPP synthase is suggested to be a key enzyme in the biosynthesis of forskolin. Herein, we report the cDNA encoding *C. forskohlii *GGPP synthase and its heterologous expression in *E. coli*.

## Results and discussion

### cDNA cloning and sequencing of *C. forskohlii GGPP synthase *gene

**Figure 1:**
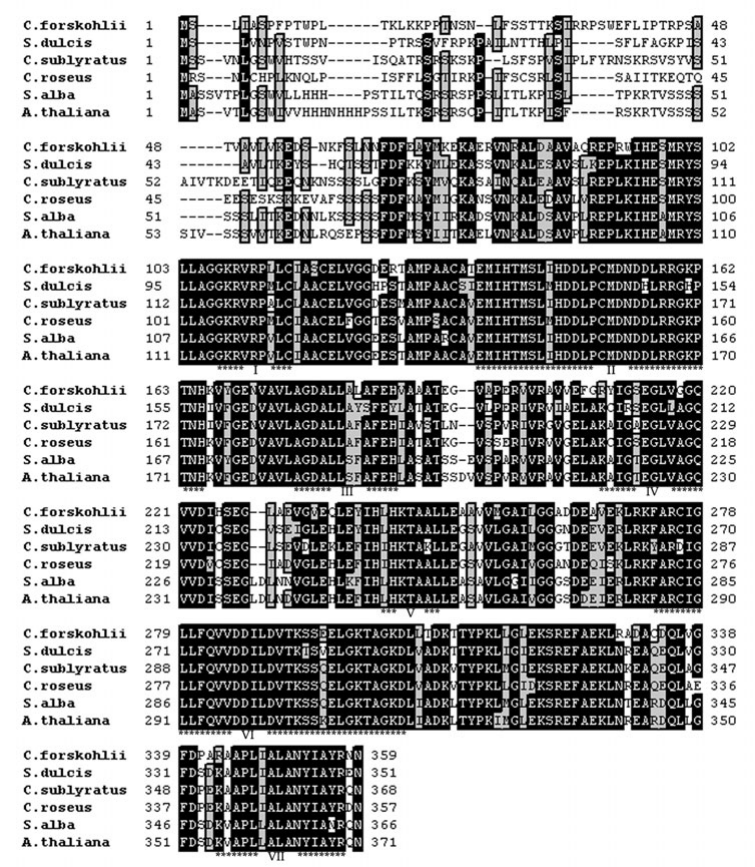
**Amino acid sequence alignment of *C. forskohlii *GGPP synthase and other plant GGPP synthases. **The residues boxed in black indicate the positions of at least five of the six compared sequences while the gray shading indicates similar amino acids. Dashes indicate gaps introduced for optimization of the alignment. Numbers of amino acids are indicated in the left and right margins. Asterisks show the seven domains that are highly conserved among prenyltransferases with two aspartate-rich motifs (domain II and VI). Species and accession numbers are *C. forskohlii*, **AY515700**; *S. dulcis*, **AB034250_1**; *C. sublyratus*, **AB034249_1**; *C. roseus*, **T09966**; *S. alba*, **T10452**; *A. thaliana*, **F85434**.

The open reading frame (ORF) for full-length *GGPP synthase *gene encodes a protein of 359 amino acids, 1,077 nucleotides long, with a calculated molecular mass of 39.3 kDa. The amino acid sequence of *C. forkohlii *GGPP synthase revealed high homology throughout the entire coding region of *Catharanthus roseus *(75%), *Arabidopsis thaliana *(73%), *Sinapis alba *(72%), *Croton sublyratus *(69%), *Scoparia dulcis *(67%) and *Mentha piperita *(64%) (Fig. 1). However, comparison of the amino acid sequence with that of prokaryotic GGPP synthases showed a low level of homology (30–53%). Highly conserved residues were designated as domains I-VII. Two conserved aspartate-rich motifs, DDXX(X)D, were identifed. FARM and SARM have been shown to be important in substrate binding and catalysis [20-22].

### Transient expression of putative localization signal of *C. forskohlii *GGPP synthase in tobacco cells

**Figure 2:**
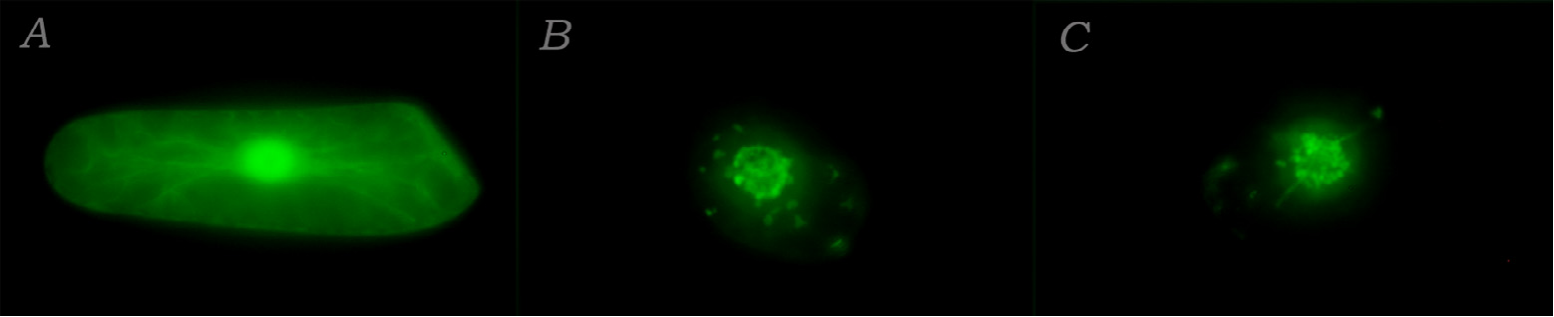
**Transient expression of GFP and GFP fusion proteins in BY-2 tobacco cells. **(A) GFP protein [35SΩ-sGFP (S65T)]; (B) *Arabidopsis *chloroplast targeting signal (pt)-GFP fusion protein [35SΩ-pt-sGFP (S65T)]; (C) putative localization signal of GGPP synthase-GFP fusion protein [35SΩ-*GGPP synthase*-sGFP (S65T)]

Sequence alignment of plant GGPP synthases showed that the N-terminal region has a low level of homology. It is reasonable to assume that these GGPP synthases have localization signals in their N-terminal regions to target them into specific subcellular compartments. The N-terminal region of *C. forskohlii *GGPP synthase was predicted to be localized in chloroplasts by the ChloroP 1.1 Prediction Server. In an effort to determine the localization of *C. forskohlii *GGPP synthase, the sequence coding for the 80 amino acid sequence at the N-terminus of *C. forskohlii *GGPP synthase was fused to the N-terminus of the GFP reporter gene and transformed into BY-2 tobacco cells. The pattern of putative localization signal of *C. forskohlii *GGPP synthase was identical to the positive chloroplast targeting signal [35SΩ-pt-sGFP(S65T)] (Fig. 2). The N-terminal region of *C. forskohlii *GGPP synthase was determined to contain a chloroplast localization signal. Recently, plant GGPP synthases have been determined to be translocated into plastids, mitochondria and cytosol [9,23].

### Heterologous expression and activity of *C. forskohlii *GGPP synthase

**Figure 3:**
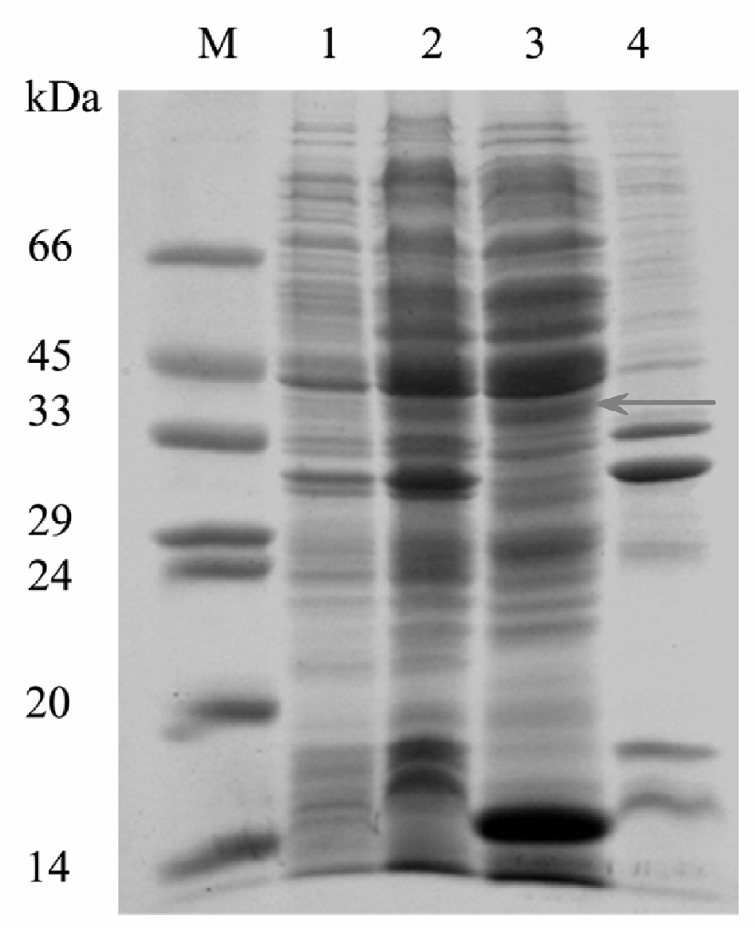
**Expression of GGPP synthase in pBluescript II KS-vector analysed by SDS-PAGE. **M = molecular mass standards are indicated in kDa; lane 1 = control (*E. coli *transformed with pBluescriptII KS^-^); lane 2 = total protein extract after 6 hours of IPTG induction; lane 3 = soluble cytoplasmic fraction of cells treated with IPTG; and lane 4 = insoluble fraction of cells treated with IPTG

In order to express *C. forskohlii GGPP synthase*, the gene was constructed and cloned into the plasmid pBluescript II KS^-^. The fusion protein of GGPP synthase with *lacZ *had a calculated molecular mass of 41.6 kDa, was observed in the soluble fraction of *E. coli *carrying pGGPPS after IPTG induction (Fig. 3).

**Figure 4:**
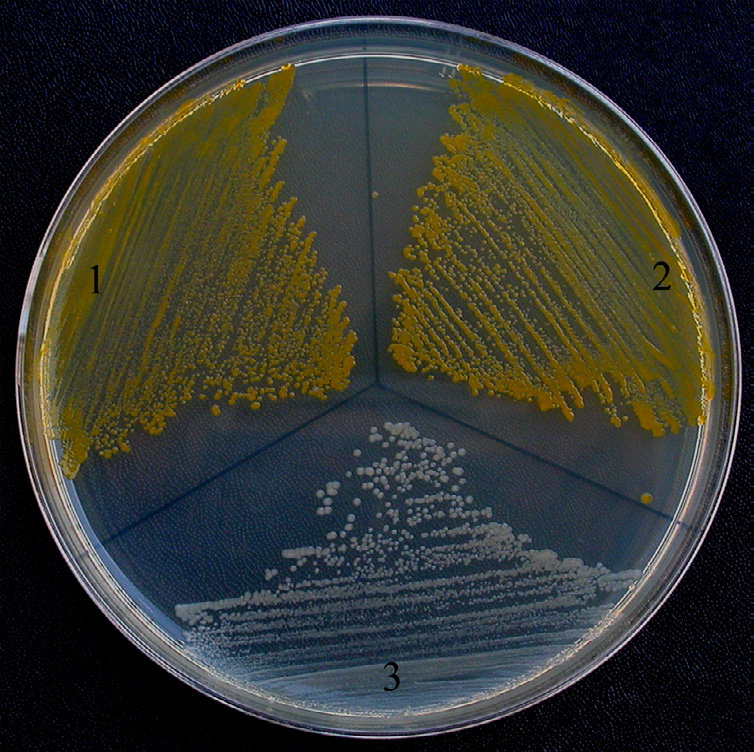
**Carotenoid production of *E. coli *harboring plasmid pACCAR25ΔcrtE and plasmid expressing. **(1) mouse GGPP synthase, pBAA; (2) plasmid expressing *C. forskohlii *GGPP synthase, pGGPPS; and (3) pBluescript II KS^- ^vector, pBS

Functional activity of expressed GGPP synthase was investigated by genetic complementation with the carotenogenic *crt *gene cluster. Carotenoids are produced in *E. coli *harbouring a *crt *cluster gene from *E. uredovora*. Replacements of a *crt *gene with an unknown gene with the same activity, can be used to determine the function of the gene [15]. Herein, the *C. forskohlii GGPP synthase *gene was cloned into pBluescript II KS^- ^vector (pGGPPS) in order to produce a *lacZ *fusion protein. pGGPPS was then transformed into *E. coli *DH10B carrying the plasmid pACCAR25Δ*crtE *in which the *crtE *encoding GGPP synthase had been deleted. The yellow color of carotenoid was observed in the transformant, indicating that pGGPPS carried the gene substituting the function of the *crtE *gene (Fig. 4). Carotenoid production of the transformants was compared with that of *E. coli *transformant carrying plasmid pACCAR25Δ*crtE *and pBAA encoding mouse GGPP synthase (positive control) [16], and with transformant carrying plasmid pACCAR25Δ*crtE *and a pBluescript II KS^- ^(pBS) vector (negative control). This result suggested that the coding region of a cDNA of *C. forskohlii GGPP synthase *encodes a functional GGPP synthase.

### Expression of *GGPP synthase *gene in organs of *C. forskohlii*

**Figure 5:**
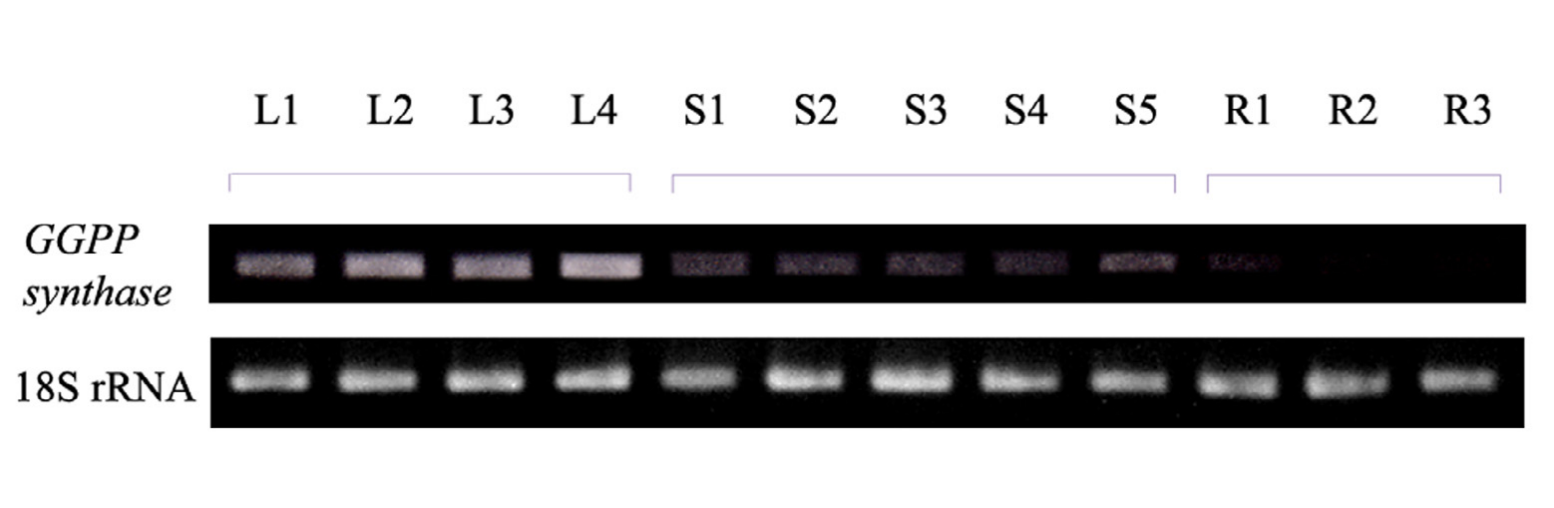
**Expression of *GGPP synthase *in roots, stems and leaves of *C. forskohlii *culture. **Ten microliters of PCR product was loaded in each lane. The lower panel shows 18S rRNA fragment as an internal control.

The expression of *GGPP synthase *gene was investigated by RT-PCR in different organs of *C. forskohlii*. Total RNA extracted from the roots, stems and leaves of an eight-month-old plant were analysed. The *C. forskohlii GGPP synthase *gene was strongly expressed in the leaves, whereas expression was decreased in stems and barely expressed in roots (Fig. 5). Therefore, the leaves are thought to be the primary location for forskolin synthesis. We previously reported the forskolin concentration in clonally propagated plant organs of *C. forskohlii *[24]. Tuberous roots and the stem base were determined to contain a higher concentration of forskolin than the organs. Moreover, the stem base, parts of the epidermis and cortex, the vascular bundle, and the pith were analysed separately. The highest concentration of forskolin was identified in the vascular bundle tissue. From these data, we proposed that GGPP synthase involved in biosynthesis of forskolin, is mainly synthesised in leaves, subsequently distributed to stems and finally accumulated in stem bases and roots.

### Forskolin production via non-mevalonate pathway

**Figure 6:**
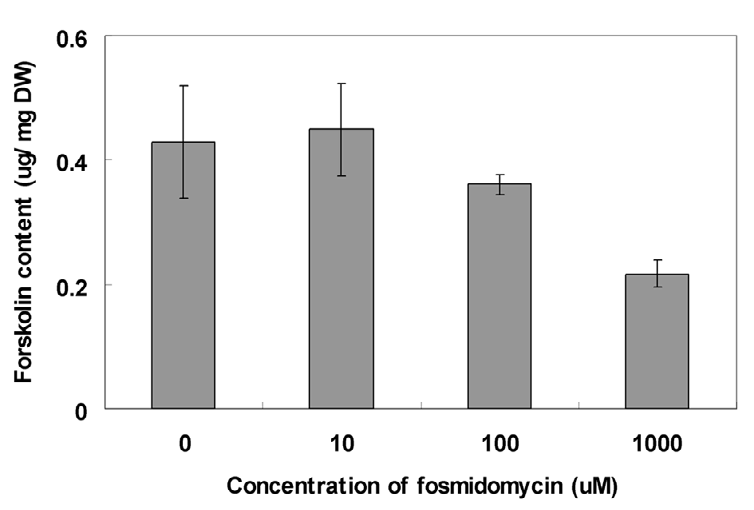
Effect of fosmidomycin on forskolin production.

In an effort to investigate the forskolin biosynthesis pathway by a non-mevalonate pathway, various concentrations of fosmidomycin, the specific inhibitor of 1-deoxy-D-xylulose-5-phosphate reductoisomerase (DXR) enzyme in the non-mevalonate pathway were applied to the *C. forskohlii *culture and the forskolin content of roots was determined (Fig. 6). Treatment led to a decrease in forskolin, whereas 10 μM fosmidomycin had no effect on forskolin production. At higher concentrations a dose-dependent inhibitory effect was observed. At 1000 μM fosmidomycin, the forskolin content was decreased by up to fifty percent in comparison to the control tissue without inhibitor treatment. Thus, forskolin was thought to be synthesised via a non-mevalonate pathway.

A recent ^13^C-glucose feeding experiment using ^13^C-NMR analytical methodology suggested the biosynthetic pathway of forskolin via a non-mevalonate pathway [25]. In addition, the DXR gene regarding the specific enzyme in the first step of the non-mevalonate pathway was cloned from *C. forskohlii *[26].

## Conclusions

*C. forskohlii *GGPP synthase was cloned and its subcellular localization was determined. The N-terminal region contained a signal which was localized in chloroplasts. Functional expression of GGPP synthase was investigated by genetic complementation with the carotenogenic *crt *gene cluster. Carotenoids were produced when the *crtE *gene was replaced with *C. forskohlii *GGPP synthase. GGPP synthase is thought to be involved in biosynthesis of forskolin, which is primary synthesised in the leaves, subsequently distributed to stems and finally accumulated in stem bases and roots.

## Methods

### Plant materials and reagents

*C. forskohlii *plantlets were cultured in hormone-free MS (Murashige and Skoog) medium at 25°C under a 16 hours light cycle. The light intensity was 3000 lux and the relative humidity was 60%. Shoot cuttings (10 mm in length) propagated by shoot tip culture were successively cultivated in vermiculite. BY-2 tobacco single cell suspension [27] was cultured in liquid modified LS (Leinsmaier and Skoog) medium supplemented with 0.2 mg l^-1 ^of 2,4-D (2,4-dichlorophonoxy acetic acid) under dark conditions at 25°C on an orbital incubator. Restriction enzymes, ligase, and PCR-polymerase were purchased from Takara Shuzo Co., Ltd. (Tokyo, Japan) and Toyobo Co., Ltd. (Tokyo, Japan). Fosmidomycin (FR-3154) was purchased from Molecular Probes (Oregon, USA). Chemical reagents were purchased from Sigma Chemical Company (St. Louis, USA) and Nacalai Tesque Inc. (Tokyo, Japan)

### Bacterial strains and plasmids

*E. coli *TOP10F' and *E. coli *DH10B carrying the plasmid pACCAR25Δ*crtE *were used in the present investigation. The pUC119 vector was used for cDNA cloning and sequencing. The pBluescript II KS^- ^vector was used as a *GGPP synthase *expression plasmid. The 35SΩ-sGFP(S65T) plasmid was used as a green fluorescent protein (GFP) reporter plasmid. The pBI121 plant vector and *Agrobacterium tumefaciens *LBA4404 were used for transformation of GFP and GFP-fusion genes to plant cells.

### cDNA cloning and sequencing of *C. forskohlii GGPP synthase *gene

Total RNA was prepared from roots of the *C. forskohlii *culture using the acid guanidium-phenol-chloroform extraction procedure [28]. Single strand cDNA was synthesised using an oligo-dT adapter primer, M-MLV reverse transcriptase and total RNA as template. Degenerate primers were designed based on highly conserved amino acid sequences of previously cloned genes encoding plant GGPP synthases [13]. A 470 bp cDNA fragment was amplified using a nested PCR with Taq DNA polymerase and degenerate primers A, B, C and D (Table 1). The 3' end of cDNA was amplified using 3' rapid amplification of cDNA ends (RACE) with gene specific primers I and J, and adapter primer F. A 522 bp product was obtained by nested PCR. For 5' RACE, the first strand cDNA was polyadenylated at its 5' end by terminal deoxynucleotidyl transferase. The first and second PCR were performed with specific primers G and H and adapter primers E and F. A 285 bp product was obtained. The entire coding region of 1,077 bp was amplified by nested PCR using specific primers K, L, M and N designed from 5' and 3' RACE products.

All amplified cDNA fragments were purified and digested with restriction enzymes at sites introduced via the PCR primers, and cloned into the vector pUC119. After transformation to *E. coli *TOP10F', clones harboring inserts were sequenced using a Model 310 Genetic Analyzer (PE Biosystems) using a BigDye Terminator Cycle Sequencing Kit.

The amino acid sequence deduced from the nucleotide sequence was compared with sequence databases in the Genome Net WWW server using the FASTA program. Multiple amino acid sequence alignment was performed using the CLUSTALW Multiple Sequence Alignment in the GenomeNet CLUSTALW Server.

### Construction and expression of putative localization signal of *C. forskohlii *GGPP synthase

A 240 bp fragment of the N-terminal region of *C. forskohlii GGPP synthase *was PCR-amplified using primers P and Q and the PCR product was digested and cloned into the *Sal*I-*Nco*I site of the 35SΩ-sGFP(S65T) plasmid. 35SΩ-pt-sGFP(S65T) was used as the positive control for chloroplast targeting [29,30]. GFP, *GGPP synthase*-GFP fusion and pt-GFP fusion with CaMV35SΩ promoter and NOS3' terminator [35SΩ-sGFP (S65T), 35SΩ-*GGPP synthase*-sGFP (S65T) and 35SΩ-pt-sGFP (S65T), respectively] were subcloned into the *Hind*III-*EcoR*I site of the pBI121 vector and then transformed into *A. tumefaciens *LBA4404. The transformants were cultured at 28°C for two days in YEB liquid medium containing 25 μg/ml of kanamycin and 25 μg/ml of rifampicin. The transformants were washed twice and re-suspended in YEB medium. *Agrobacterium *transformants (10^8 ^cells) were applied to four ml of five-day-old BY-2 suspension culture. The culture was incubated at 28°C for two days under dark conditions. GFP and GFP fusion protein were analysed by fluorescence microscopy using Nikon Eclipse TE2000-U model. Cells were observed at a 400 × magnification.

### Construction of plasmid for *C. forskohlii *GGPP synthase expression

The coding region of a cDNA of *C. forskohlii GGPP synthase *was amplified by PCR using specific primers M and O. A PCR product was digested; purified and cloned into the *Kpn*I*-Sal*I site of pBluescript II KS^- ^vector, namely pGGPPS. This plasmid was transformed into *E. coli *XL1-Blue MRF' for over-expression. The transformants were cultured in LB liquid medium containing 50 μg/ml of ampicillin and 25 μg/ml of chloramphenicol. The culture was induced with 1 mM isopropyl-1-thio-β-D-galactoside (IPTG) and incubated for six hours at 37°C. The cells were harvested and washed with 50 mM Tris-HCl pH 8.0 by centrifugation. The pellet was re-suspended, lysozyme was added and the mixture was incubated for 30 minutes. The mixture was then sonicated for four cycles of 15 seconds at one minute intervals. The soluble fraction was obtained after centrifugation at 10,000 × g for 10 minutes. SDS-PAGE was conducted in order to detect the proteins [31].

### Genetic complementation expression

The pACCAR25Δ*crtE *plasmid contains the gene cluster *crtB*, *crtI*, *crtX*, *crtY *and *crtZ *encoding carotenoid biosynthetic enzymes with the exception of *crtE *(encoding GGPP synthase). The plasmid pBAA containing mouse *GGPP synthase *(positive control plasmid) and *E. coli *DH10B carrying the plasmid pACCAR25Δ*crtE *was provided by Dr. M. Kawamukai, Shimane University, Japan [16]. pBluescript II KS^- ^vector, pBS, was used as negative control. pGGPPS, pBAA and pBS were transformed into *E. coli *DH10B carrying the plasmid pACCAR25Δ*crtE*. All transformants were plated on LB agar medium containing 50 μg/ml of ampicillin and 25 μg/ml of chloramphenicol and then incubated for two to three days at 25°C.

### Reverse transcriptase-PCR (RT-PCR)

An eight-month-old *C. forskohlii *was analysed in twelve separate parts; leaf (L1–L4), stem (S1–S5) and root (R1–R3). The numbering is based on the maturation of organs. Total RNA was extracted from each part of plant. One microgram of total RNA was used as the template for the synthesis of the first strand cDNA (using SuperScript First-Strand Synthesis System for RT-PCR, Invitrogen). Primers M and O, the first strand cDNA and KOD-polymerase were used for the amplification of *C. forskohlii GGPP synthase *with the condition of denaturation, 98°C, 15 seconds; annealing, 60°C, 2 seconds and extension, 74°C, 5 seconds. The 18S rRNA fragment used as an internal control was amplified using primers R and S under the same conditions of *C. forskohlii GGPP synthase *amplification. The amplified PCR products were analysed by 1.0% agarose gel electrophoresis.

### Analysis of forskolin production

*C. forskohlii *plantlets were treated with various concentrations of fosmidomycin and then investigated for forskolin content using the HPLC method as previously described [26]. Forskolin was detected by comparison with the retention time of a forskolin standard (Sigma) detected by UV absorption at 202 nm.

## List of abbreviations

*crt*, carotenogenic gene; FARM, first aspartate-rich motif; GFP, green fluorescent protein; GGPP, geranylgeranyl diphosphate; MEP, 2C-methyl-D-erythritol 4-phosphate; MVA, mevalonate; SARM, second aspatate-rich motif

## Authors' contributions

SE carried out the molecular genetic studies, participated in the sequence alignment, forskolin analysis and drafted the manuscript. TF participated in the design of the study and coordination. MK participated in genetic complementation and coordination. YS conceived the study and participated in its design and coordination. All authors read and approved the final manuscript.

## Figures and Tables

**Table 1:** Primers for cDNA cloning of *GGPP synthase*.

Primers	Sequences (5' to 3')	Designed from
A	GGGGTACCYITTGYATHGCIGCITAYGA	conserved sequence (LCIAACE)
B	GGGGTACCGTIGARATGATHCAYACIAT	conserved sequence (VEMIHTM)
C	CGGGATCCTTIGTIACRTCIARDATRTC	conserved sequence (DILDVTK)
D	CGGGATCCARRTCYTTICCIGCIGTYTT	conserved sequence (KTAGKDL)
E	GACTCGTCTAGAGGATCCCGT	adapter primer
F	GACTCGTCTAGAGGATCCCGT_17_	oligo-dT adapter primer
G	GGGGTACCCCTTATGGTTGGTGGGCTTC	degenerated PCR product (KPTNHK)
H	CGCCACGTTCTCGCCGTAGA	degenerated PCR product (VYGENVA)
I	CCATATTGGGTGGCGCCGAT	degenerated PCR product (AILGGAD)
J	GGGGTACCGATGAGGCGGTGGAGAAGCT	degenerated PCR product (DEAVEKL)
K	ATTTGGTGTTCGACGACGAC	5'RACE
L	GGGGTACCCATATGAGCCTCATCGCGAGTC	5'RACE
M	GCACGCGTCGACATTGTTCCGGTAAGCAAT	3'RACE
N	AGTGAAATGGAAATTATTCA	3'RACE
O	GGGGTACCGATGAGCCTCATCGCGAGTCCA	5'RACE
P	GCACGCGTCGACAACAATGAGCCTCATCGCG	N-terminal sequence (MSLIAS)
Q	CATGCCATGGGGTTCACCCGCTCTGCCTTCT	N-terminal sequence (EKAERVN)
R	TTCTTGGATTTATGAAAGACGAACAAC	18S rRNA
S	AAGACCAACAATTGCAATGATCTATCC	18S rRNA
